# Supplementary figures and images for: Stress adaptive plasticity from Aegilops tauschii introgression lines improves drought and heat stress tolerance in bread wheat (Triticum aestivum L.)
Source: PeerJ. 2024 Jun 11;12:e17528. doi: 10.7717/peerj.17528 (PMC11177856; doi:10.7717/peerj.17528)

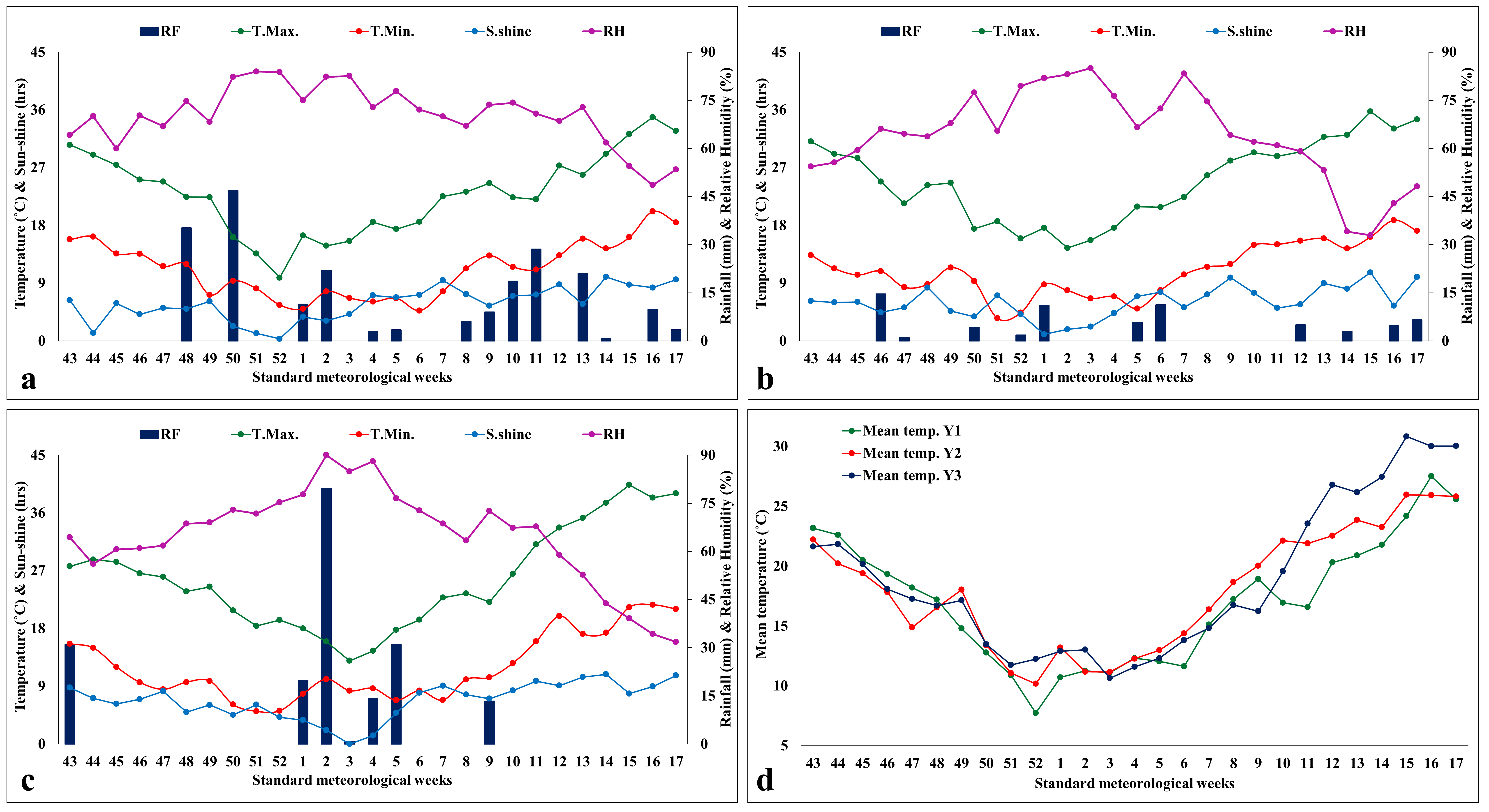

Supplement: Supplemental Information 2 — (a-c) mean maximum and minimum temperatures (° C), total rainfall (mm), mean sun-shine hours (hours/day), and mean relative humidity (%) for 2019-20 (a), for 2020-21 (b), for 2021-22 (d); (d) mean temperature for 2019-20, 2020-21, and 2021-22 [file peerj-12-17528-s002.png]

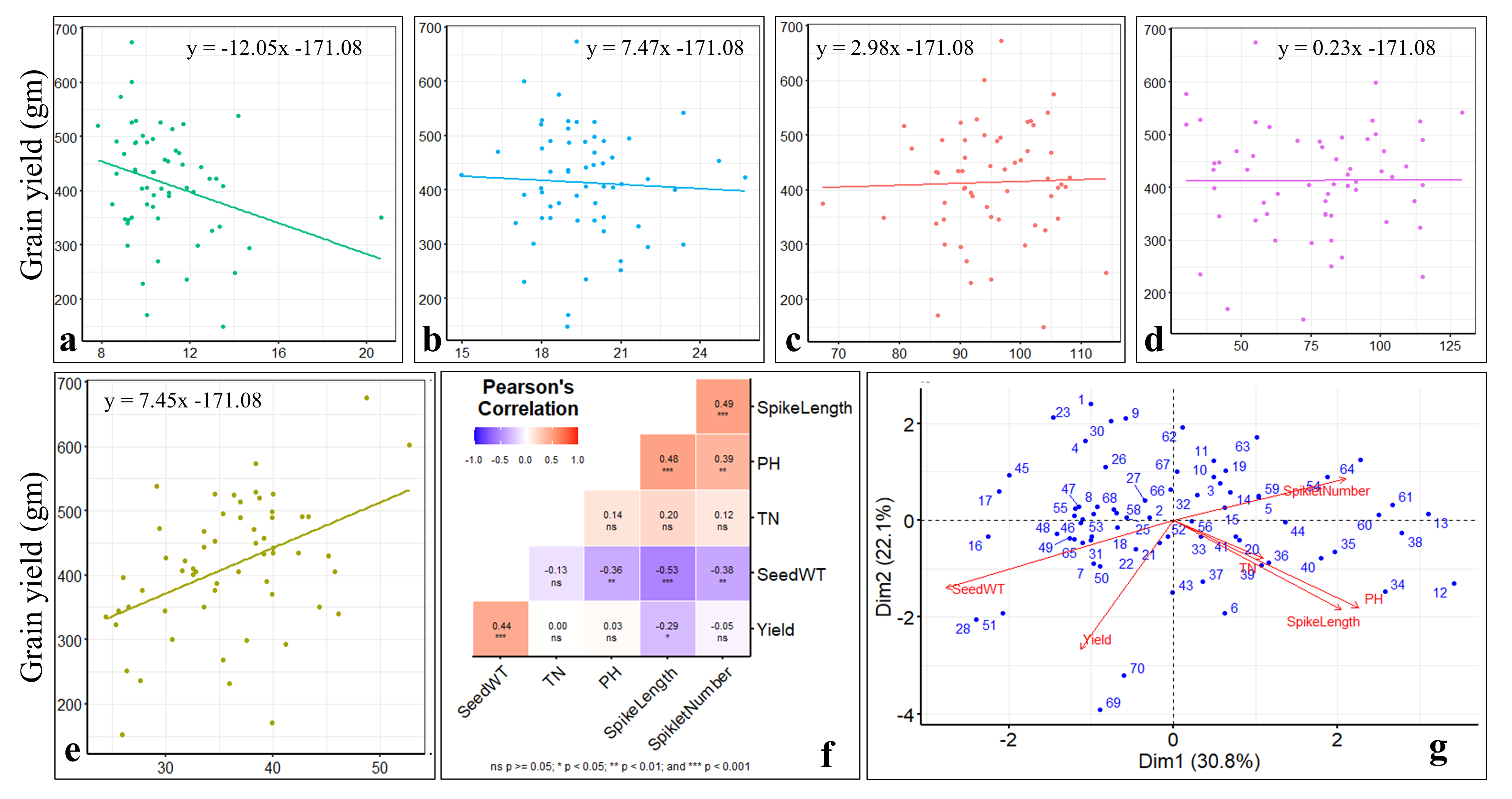

Supplement: Supplemental Information 3 — (a-e) linear regression analysis of grain yield with spike length (a), spikelet number (b), plant height (c), tiller number (d), 1000 seed weight (e); (f) Pearson’s correlation between different agronomic traits; (g) principal component analysis for agronomic traits. [file peerj-12-17528-s003.png]

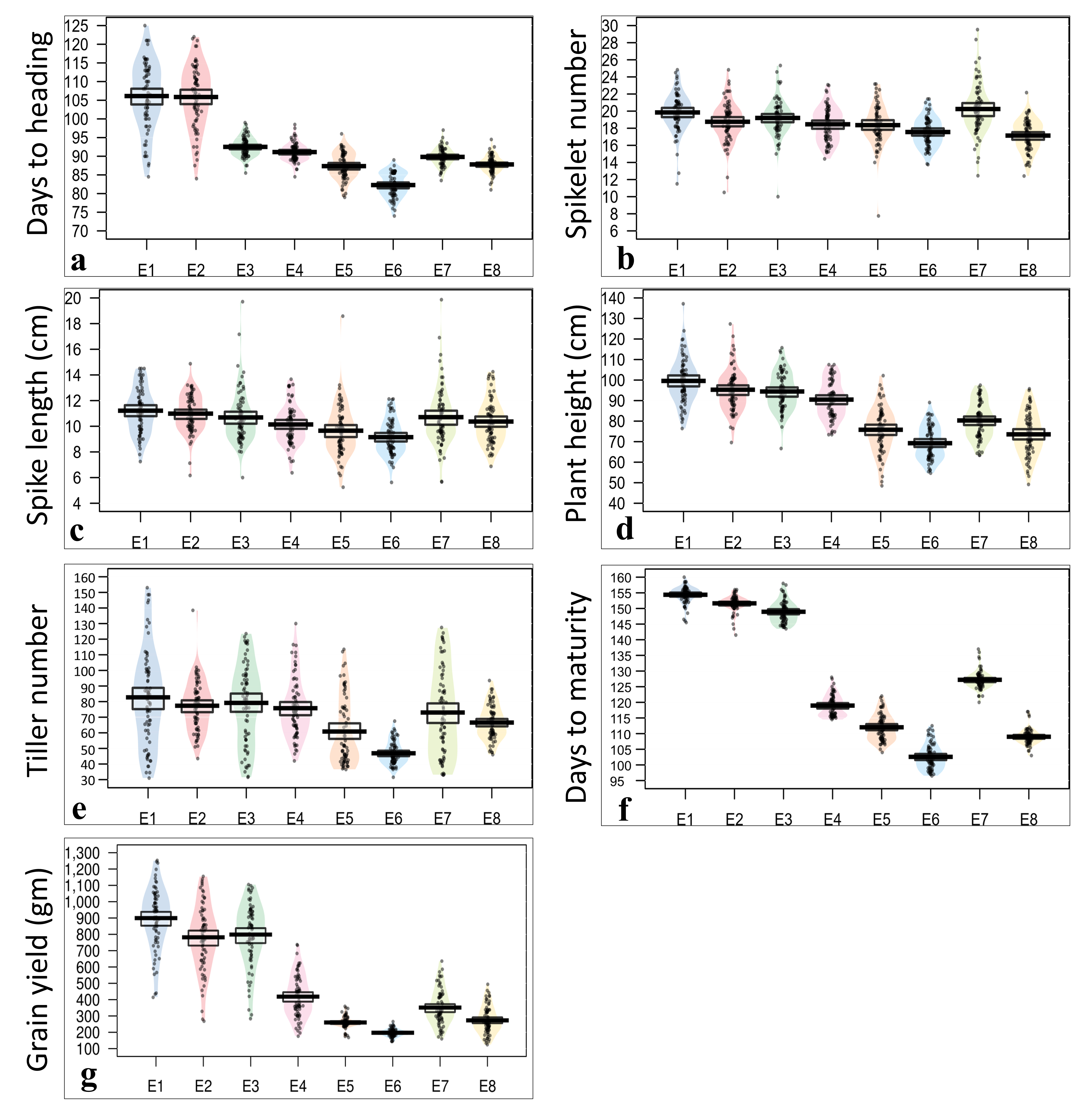

Supplement: Supplemental Information 4 — (a) days to heading; (b) spikelet number; (c) spike length; (d) plant height; (e) tiller number; (f) days to maturity; and (g) grain yield (gm/plot) over 2020-21 and 2021-22. E1: early sowing in 2020-21; E2: early sowing in 2021-22; E3: timely sowing in 2020-21; E4: timely sowing in 2021-22; E5: late sowing in 2020-21; E6: late sowing in 2021-22; E7: rainfed sowing in 2020-21; E8: rainfed sowing in 2021-22. [file peerj-12-17528-s004.png]

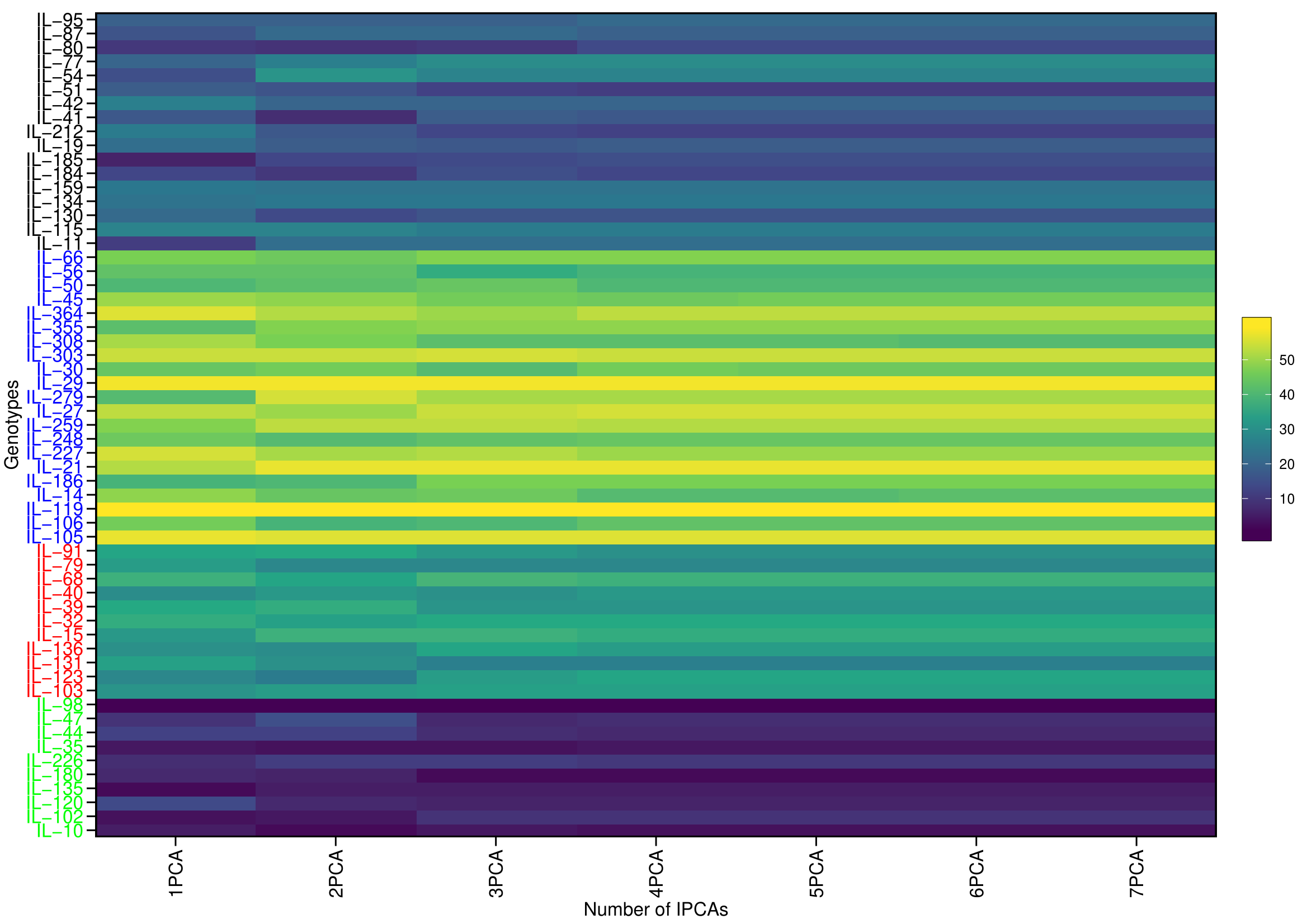

Supplement: Supplemental Information 5 [file peerj-12-17528-s005.png]

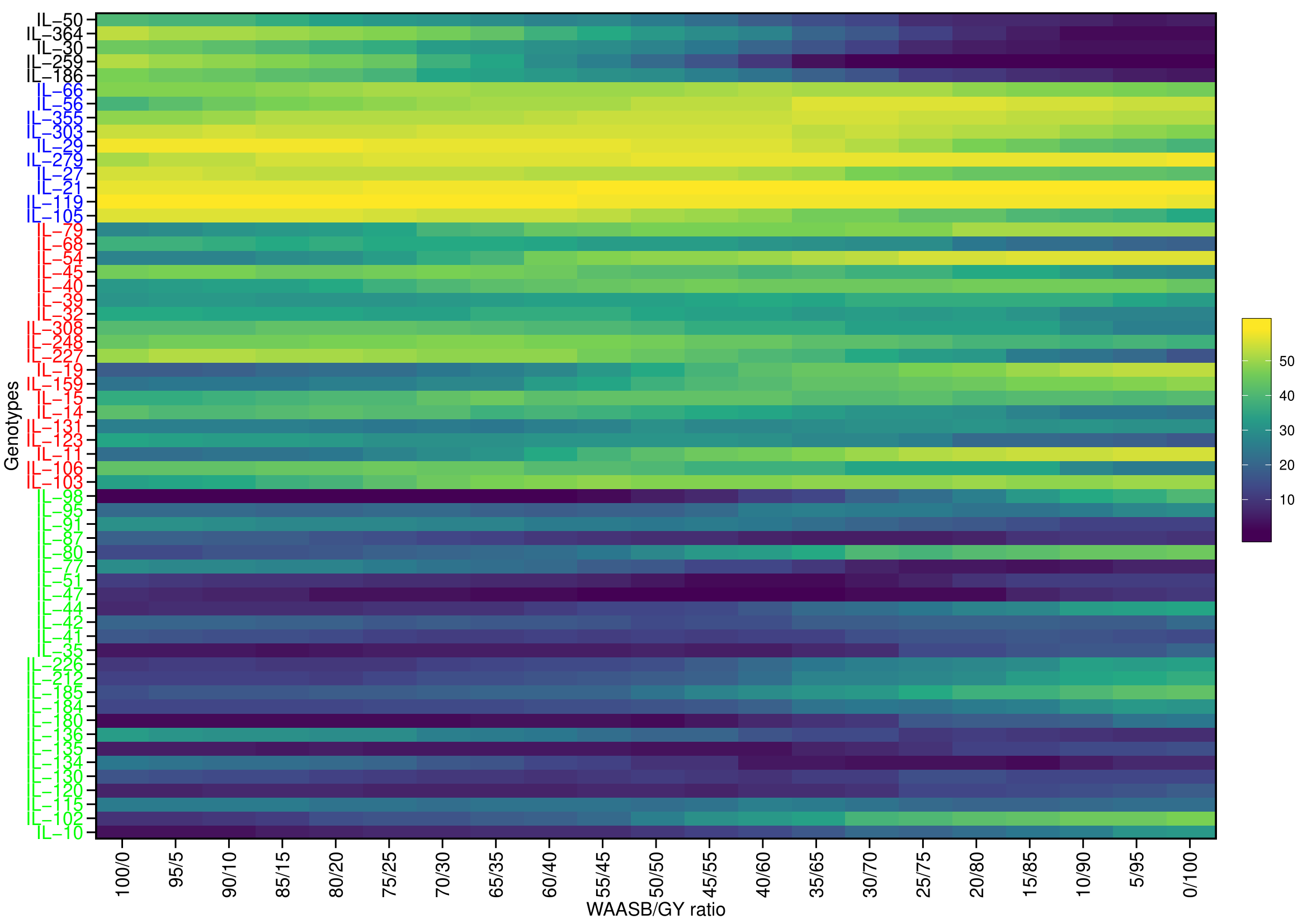

Supplement: Supplemental Information 6 [file peerj-12-17528-s006.png]
