# Supplementary material for: Stress adaptive plasticity from Aegilops tauschii introgression lines improves drought and heat stress tolerance in bread wheat (Triticum aestivum L.)
Source: PeerJ. 2024 Jun 11;12:e17528. doi: 10.7717/peerj.17528 (PMC11177856; doi:10.7717/peerj.17528)
Supplement: Supplemental Information 14 [file peerj-12-17528-s014.docx]

Dear PeerJ,

Subject: Adding additional author to manuscript submitted in PeerJ.

I hope this letter finds you well. I am writing to request add **Mohit Jain** to a manuscript currently under consideration for publication in PeerJ journal, entitled "Stress adaptive plasticity from *Aegilops tauschii* in introgression lines improves drought and heat stress tolerance in bread wheat (*Triticum aestivum* L.)”.

Mohit Jain has made significant contributions to the research by collecting various agronomic and physiological data in field (during 2020-21 and 2021-22) and laboratory (during 2021). Additionally, he is involved in preparing attractive figures and tables for the manuscript. However, in the initial submission, by chance/mistake we missed adding his name in the manuscript. We believe that his contributions and involvement in the project permit authorship.

Below are the details of the proposed additional author:

Name: Mohit Jain

Affiliation: Department of Plant Breeding and Genetics, Punjab Agricultural University, Ludhiana, India

Email: [mohit.jn1997@gmail.com](mailto:mohit.jn1997@gmail.com)


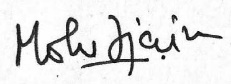


Signature (Mohit Jain)

Further, all co-authors of this manuscript are agreed and signed (see the table below) to add Mohit Jain as a co-author for this manuscript.

| **Name of author** | **Designation with email ID** | **Signature** |
| --- | --- | --- |
| Dr. Santosh Gudi | Department of Plant Breeding and Genetics, Punjab Agricultural University, Ludhiana, India  Email ID: [santosh-pbg@pau.edu](mailto:santosh-pbg@pau.edu) | 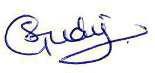  03/30/2024 |
| Dr. Satinder Singh | Department of Plant Breeding and Genetics, Punjab Agricultural University, Ludhiana, India  Email ID: [satinder-pbg@pau.edu](mailto:satinder-pbg@pau.edu) | 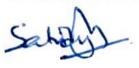  03/28/2024 |
| Dr. Satinder Kaur | School of Agricultural Biotechnology, Punjab Agricultural University, Ludhiana, Punjab, India  Email ID.: [satinder.biotech@pau.edu](mailto:satinder.biotech@pau.edu) | 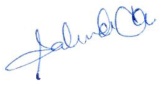  03/28/2024 |
| Dr. Puja Srivastava | Department of Plant Breeding and Genetics, Punjab Agricultural University, Ludhiana, India  Email ID: [pujasrivastava@pau.edu](mailto:pujasrivastava@pau.edu) | 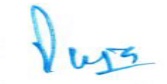  03/28/2024 |
| Dr. Gurvinder Singh Mavi | Department of Plant Breeding and Genetics, Punjab Agricultural University, Ludhiana, India  Email ID: [G.S.Mavi@pau.edu](mailto:G.S.Mavi@pau.edu) | 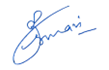  03/28/2024 |
| Dr. Parveen Chhuneja | School of Agricultural Biotechnology, Punjab Agricultural University, Ludhiana, Punjab, India  Email ID: [pchhuneja@pau.edu](mailto:pchhuneja@pau.edu) | 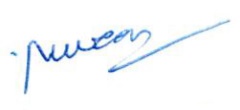  03/28/2024 |
| Dr. Virinder Singh Sohu | Department of Plant Breeding and Genetics, Punjab Agricultural University, Ludhiana, India  Email ID: [sohuvs@pau.edu](mailto:sohuvs@pau.edu) | 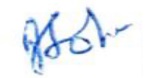  03/28/2024 |
| Dr. Fatmah Ahmed Safhi | Department of Biology, Princess Nourah bint Abdulrahman University, Riyadh, Saudi Arabia  Email ID: [faalsafhi@pnu.edu.sa](mailto:faalsafhi@pnu.edu.sa) | 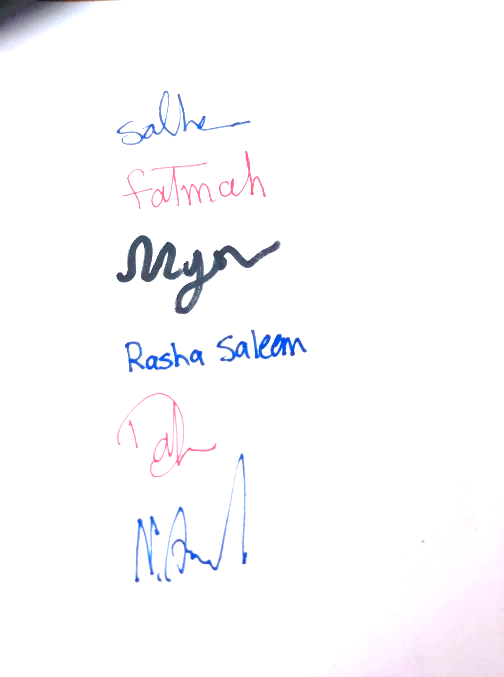  03/29/2024 |
| Dr. Diaa Abd El Moneim | Department of Plant Production (Genetic Branch), Faculty of Environmental Agricultural Sciences, Arish University, Egypt  Email ID: [dabdelmoniem@aru.edu.eg](mailto:dabdelmoniem@aru.edu.eg) | 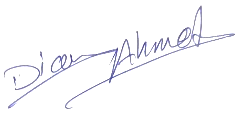  03/29/2024 |
| Dr. Achla Sharma | Department of Plant Breeding and Genetics, Punjab Agricultural University, Ludhiana, India  Email ID: [achla12@gmail.com](mailto:achla12@gmail.com) | 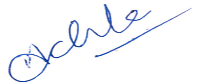  03/30/2024 |

We kindly request you to consider our request.

Thank you for your attention to this matter. We look forward to your favorable consideration.


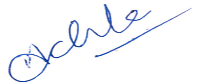
Sincerely,

Dr Achla Sharma

Principal Wheat Breeder

Punjab Agricultural University, Ludhiana, India
